# Structural, Morphological, Optical and Photocatalytic Properties of Y, N-Doped and Codoped TiO_2_ Thin Films

**DOI:** 10.3390/ma10060600

**Published:** 2017-05-31

**Authors:** Zeineb Hamden, David Conceição, Sami Boufi, Luís Filipe Vieira Ferreira, Soraa Bouattour

**Affiliations:** 1 Faculty of Science, LCI, University of Sfax, BP1171-3018 Sfax, Tunisia; zeineb.hamden@yahoo.fr; 2 Centro de Química-Física Molecular and Institute of Nanoscience and Nanotechnology, Instituto Superior Técnico, University of Lisbon, 1049-001 Lisbon, Portugal; david.conceicao@tecnico.ulisboa.pt; 3 Faculty of Science, LSME, University of Sfax, BP1171-3018 Sfax, Tunisia; sami_boufi@yahoo.com

**Keywords:** titanium dioxide, photochemistry, band-gap engineering, photocatalysis

## Abstract

Pure TiO_2_, Y-N single-doped and codoped TiO_2_ powders and thin films deposited on glass beads were successfully prepared using dip-coating and sol-gel methods. The samples were analyzed using grazing angle X-ray diffraction (GXRD), Raman spectroscopy, time resolved luminescence, ground state diffuse reflectance absorption and scanning electron microscopy (SEM). According to the GXRD patterns and micro-Raman spectra, only the anatase form of TiO_2_ was made evident. Ground state diffuse reflectance absorption studies showed that doping with N or codoping with N and Y led to an increase of the band gap. Laser induced luminescence analysis revealed a decrease in the recombination rate of the photogenerated holes and electrons. The photocatalytic activity of supported catalysts, toward the degradation of toluidine, revealed a meaningful enhancement upon codoping samples at a level of 2% (atomic ratio). The photocatalytic activity of the material and its reactivity can be attributed to a reduced, but significant, direct photoexcitation of the semiconductor by the halogen lamp, together with a charge-transfer-complex mechanism, or with the formation of surface oxygen vacancies by the N dopant atoms.

## 1. Introduction

Nowadays, organic pollutants produced by some industries are harmful to human health and living creatures. Owing to the urgent need for a clean and comfortable environment, photocatalysis offers great potential for the elimination of toxic chemicals in the environment through its efficiency and broad applicability.

Among many semiconductors and photocatalysts, titanium dioxide TiO_2_, in the form of thin films, powders or nanostructured layers, is close to be an ideal bench mark photocatalyst in the environmental photocatalysis applications, due to its many desirable properties such as inexpensive, readily available, biologically and chemically inert, and good photoactivity [1,2]. However, the application of TiO_2_ is limited by problems associated with the fast charge (electron/hole) recombination phenomenon, and the band gap belonging to the UV region, since the absorption behavior and the separation efficiency of electron–hole pairs are two essential factors on which the photodegradation of TiO_2_ strongly depends [3]. Thus, several approaches have been used in order to overcome these difficulties, including doping with other species [4,5]. Between many chemical elements used as dopants for TiO_2_, rare earth elements such as (Y, Eu, Er, Nd…) have been widely studied [6,7]. Doping TiO_2_ with rare earth elements is employed to enhance the photochemical activity regarding degradation of organic pollutants in aqueous media and to shift the irradiation wavelength from the UV to the visible range. Narayan et al. [8] have studied the effect of rare earth (Y, Yb, Gd) ions on TiO_2_ properties using a co-precipitation/hydrolysis method and its photocatalytic activity was evaluated for the degradation of Congo red under visible light irradiation. They demonstrated that Y modified TiO_2_ provided the best photocatalytic activity due to the smaller particle size of photocatalyst and effective separation of electron–hole pairs. Wang et al. [9] synthesized also yttrium doped TiO_2_ through the sol–gel method and reported that Y-TiO_2_ has about 1.50 times greater photocatalytic activity compared to undoped TiO_2_ for the methyl orange degradation. Moreover, in a previous research work, Bouattour et al. [10], studied the photocatalytic activity of nanopowders of yttrium doped titania using sol–gel, solid state process, and 2-naphthol as a pollutant model, under sunlight irradiation. Results showed a great enhancement of the photocatalytic efficiency with the incorporation of Y in samples prepared by solid grinding. Kallel et al. [11] has studied Rb-Y codoped TiO_2_. They demonstrated that, according to the XPS and XRD analysis, the Y^3+^ dopant did not enter the TiO_2_ crystal lattice to substitute for Ti^4+^. It was dispersed uniformly onto TiO_2_ nanoparticles. Wu et al. [12] concluded in their studies that the incorporation of interstitial boron dopants would create oxygen vacancies (Ov¨) and reduce Ti^4+^ to Ti^3+^ to form the [Ov¨-Ti^3+^]+ complex, which would then trap the excited charge carriers and prolong carrier lifetime. Moreover, Y^3+^ ions could also trap the photo-excited electrons to form Y^2+^ ions, which would then react with the absorbed O2 on TiO_2_ surface to generate reactive species. On the other hand, research on the incorporation of non-metal ions (N, C, S, F) into TiO_2_ has increased since few years [13], for the reason that doping with these atoms can largely enhance the photoactivity efficiency of TiO_2_. However, there are still many controversies on the modification of TiO_2_ by these species, especially the nitrogen, since different hypotheses concerning the state of nitrogen in the N-TiO_2_ lattice and the mechanism of band gap modification have been derived. For example, Asahi et al. [4] proposed that substitutional-type doping using N was effective for the band gap narrowing of TiO_2_ due to the mixing of N 2p with O 2p states in the valence band based on spin-restricted local density approximation calculations on the anatase phase. However, Irie et al. [14] confirmed that interstitial-type doping of N atoms was related to the photothreshold energy decrease, which induced localized N 2p states within the band gap just above the top of the valence band, facilitating the production of oxygen vacancies. A third research group suggest that the band edge narrowing can occur when doping levels reach a critical concentration that exceeds 20% in anatase TiO_2_ [13]. From the above-mentioned references, it appears that the effect of dopants on the structural properties and the photocatalytic activity of TiO_2_ is a complex problem that depends not only on the dopant nature but also on the preparative procedure of the materials and the pollutant to degrade. Thus, we have considered that studying codoped TiO_2_ thin films with N as one of the dopants should be interesting to try to identify the effect of the each dopant on the properties and TiO_2_ performance. In this context, we have prepared a series of Li-, N-doped and Li-N codoped TiO_2_ thin films and powders [15]. We have demonstrated that the energy gap increases and the electron/hole recombinations are delayed for all the samples. An Li-N codoped sample exhibits interesting photoactivity compared to undoped and Li-N monodoped TiO_2_ under visible light irradiation using aromatic amines as pollutants models. In the present work, Y-N doped or codoped titania thin films with controlled composition were prepared by the sol-gel process using the dip-coating method. The effect of Y and N dopants on the structure and the phase stability was studied by X-ray diffraction and Raman spectroscopy. The efficiency of these samples as photocatalysts was investigated for the degradation of toluidine as organic compound models under a halogen lamp used as an irradiation source.

## 2. Results and Discussion

### 2.1. SEM Analysis 

**Figure 1:**
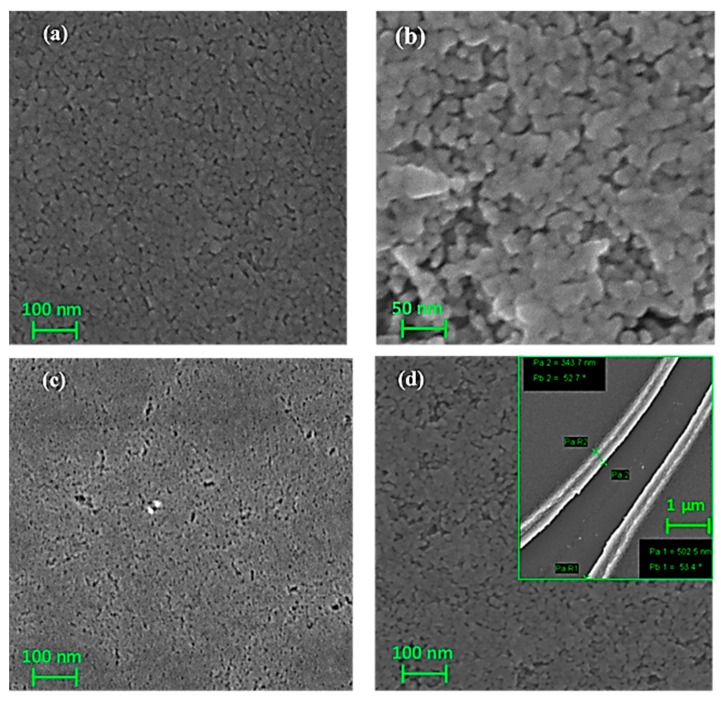
FE-SEM images of: (**a**) undoped TiO_2_ (**b**): 2% N dopedTiO_2_ (**c**): 2% Y doped TiO_2_ and (**d**): 2% Y-N codoped TiO_2_ thin films annealed at 400 °C.

Surface morphology of pure TiO_2_, single doped and Y-N codoped TiO_2_ thin film, calcined at 400 °C, is shown in Figure 1a–d. The undoped and N doped TiO_2_ was already fully characterized [14]. As can be seen from these FE-SEM micrographs, relatively dense, uniform, and nano-granular film was obtained for all samples. Higher magnification revealed a nanostructured films composed with relatively uniform shape nanoparticles with average grain size between 20 and 30 nm as estimated from scratched film (see inset Figure 1d). The thickness of film was in the range of 300–500 nm as estimated from the edge as shown in Figure 1d.

### 2.2. GXRD Characterization

**Figure 2:**
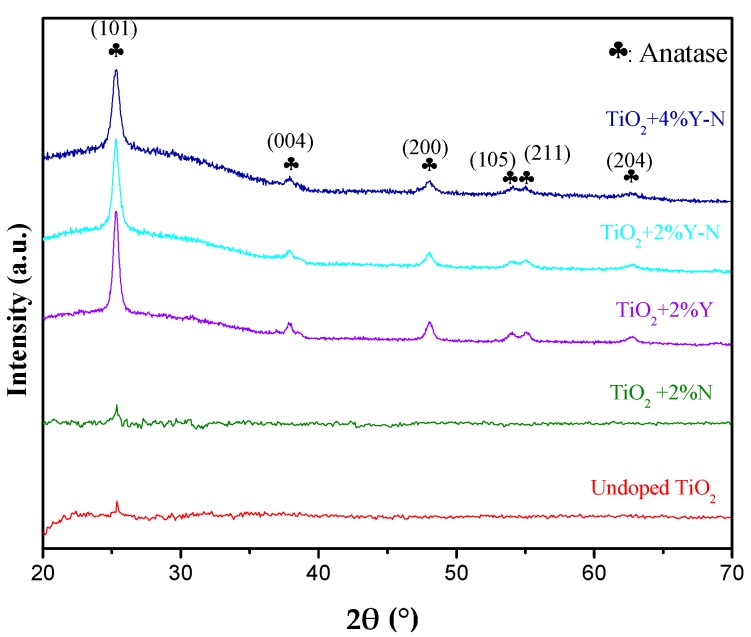
GXRD patterns of the undoped TiO_2_ and 2%, 4% Y and/or N doped TiO_2_ thin films annealed at 400 °C.

Grazing angle X-ray diffraction (GXRD) was carried out to investigate the effect of nitrogen and yttrium doping on the crystal structure of TiO_2_. Figure 2 shows the GXRD patterns of undoped TiO_2_, N-TiO_2_, Y-TiO_2_, and Y-N TiO_2_ thin films calcined at 400 °C. From these patterns, it is clear that the diffractogram recorded for the undoped TiO_2_, is slightly similar to that of TiO_2_ doped with 2% N. For both, only one weak reflection associated to the anatase phase was observed, indicating their low crystallinity. However, introducing Y as dopant improves the crystallinity of TiO_2_. Indeed, five reflections are observed for Y-TiO_2_, 2% Y-N TiO_2_ and 4% Y-N TiO_2_ samples. They appear at 2θ values: 25.3°, 37.9°, 48.03°, 53.9°, 55.1° and 62.7°, corresponding to the (101), (004), (200), (105), (211) and (204) planes, respectively. All of them are associated to the anatase phase. No reflection related to a secondary phase due to Y or N dopants is distinguished. This result is in agreement with those reported by Khan and Cao [16] and Zhang et al. [17], showing that only the anatase phase is obtained for TiO_2_ powders doped TiO_2_ using various Y concentrations. On the other hand, a careful analysis of the GXRD patterns reveal a slight refection broadening with increasing Y-doping concentration, suggesting a systematic decrease in the grain size. Applying Scherrer formula [18] and taking into account the full width at half maximum (FWHM) for the (101) reflection, typical values of crystallite sizes are calculated from XRD patterns (Figure 2). The average sizes of 2% Y doped TiO_2_, 2% Y-N codoped TiO_2_ and 4% Y-N codoped TiO_2_ particles, calcinated at 400 °C, are found to be 18, 16 and 14 nm, demonstrating that doping TiO_2_ with Y^3+^ or N contributed to lowering the size of the crystallite, therefore inhibiting the growth of TiO_2_ particle. Furthermore, it is worth to note that the presence of Y enhanced the crystallization of the TiO_2_ phase. However, it is important to be precise that these two factors: the presence of anatase phase and the decrease of particles nanosize are favorable to the photocatalytic activity.

**Figure 3:**
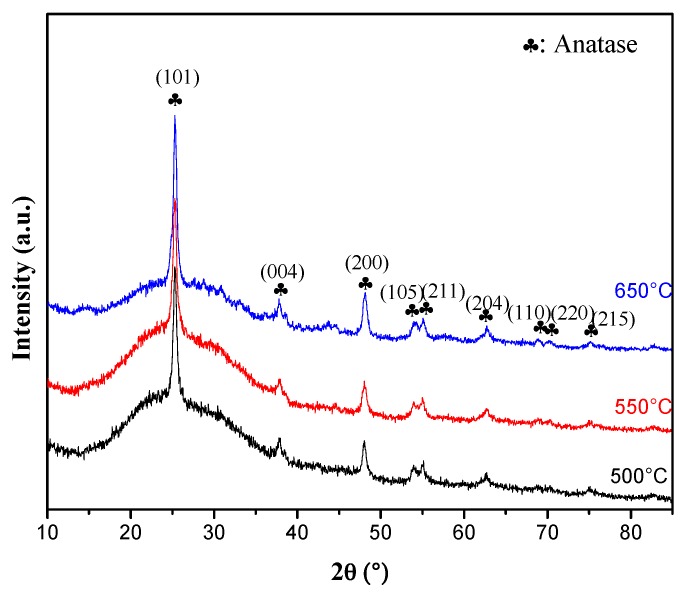
GXRD patterns of the 2% Y-N codoped TiO_2_ thin films annealed at different temperature.

Figure 3 shows the GXRD patterns of 2% Y-N codoped TiO_2_ thin film calcined at different temperatures. Only the anatase phase is observed for the samples calcined at 500 °C, 550 °C and 650 °C. This result suggests that the typical anatase-rutile phase transformation [19] is delayed to higher temperature. The anatase phase is probably stabilized by the surrounding rare earth ions through the formation of Ti-O-rare earth bonds [20].

### 2.3. Raman Spectroscopy

**Figure 4:**
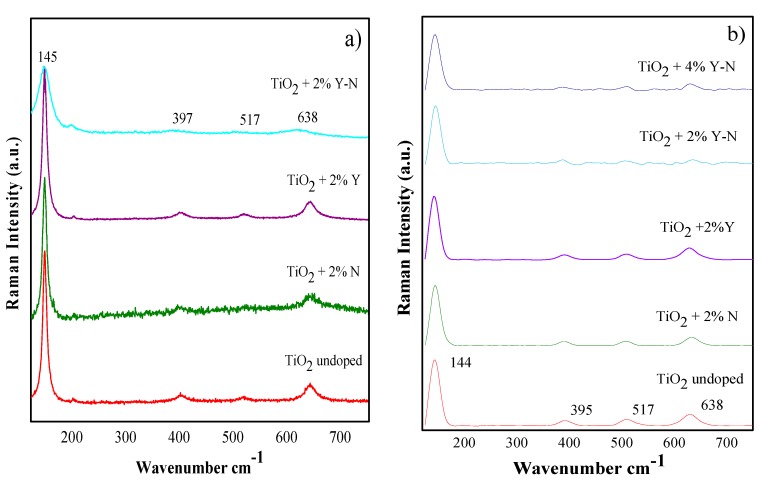
Raman spectra of undoped and 2% Y-N doped and codoped TiO_2_ thin films (**a**) and powder (**b**) annealed at 400 °C.

The structural characteristics and phase composition of the samples were further investigated by micro-Raman spectroscopy. Figure 4a,b shows the Raman spectra of undoped TiO_2_ and Y-N-doped or codoped TiO_2_, films and powders, respectively. Only the anatase phase, which is characterized by six Raman active modes (A_1g_ + 2B_1g_ + 3Eg) [21], is observed for all samples. The dominant E_g_ observed at 144 cm^−1^ is associated with the Ti-O bending vibration. It is also attributed to the two weak bands at 197 and 638 cm^−1^. However, B_1g_ and the (A_1g_ + B_1g_) bands are shown around 395 cm^−1^ and 517 cm^−1^, respectively. Furthermore, data in Figure 4a indicated a Raman band broadening for Y-N codoped TiO_2_. These results can be ascribed to phonon confinement and surface strain effects, usually observed in nanostructured materials [22]. These outcomes are in good agreement with values of crystallite size determined using Scherrer’s formula and XRD studies.

Additionally, micro-Raman analyses for the nanopowders in Figure 4b show that the introduction of N decreases the anatase crystallinity by means of a broadening effect mainly observed on the 395, 517 and 638 cm^−1^ peaks of the Raman signal. The anatase structure is greatly affected by the codoping with 2% Y-N and 4% Y-N, and it does not change with the introduction of Yttrium as a single dopant. 

### 2.4. Ground State Diffuse Reflectance Spectra

**Figure 5:**
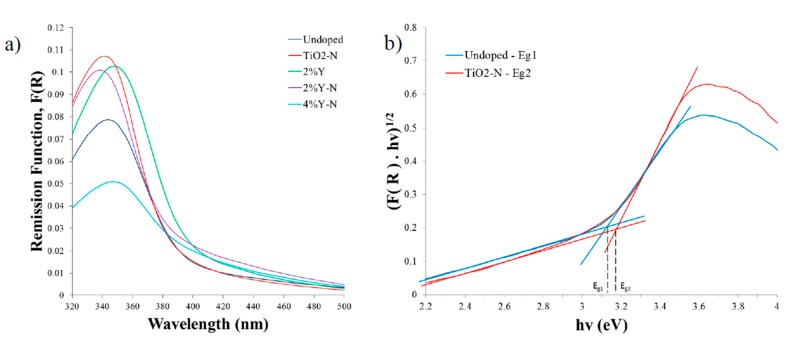
(**a**) ground-state diffuse reflectance absorption spectra of undoped, doped and codoped TiO_2_ with N and Y, annealed at 400 °C; (**b**) Tauc plots of pure and N-doped TiO_2_ samples, evidencing the calculation of the band gap energies for these two samples.

In Figure 5, one can see the ground state diffuse reflectance spectra of samples with different dopants. TiO_2_ undoped nanoparticles spectra were also included for comparison. A significant tail was observed in all diffuse reflectance spectra, reaching the visible range of wavelengths. The cut-off wavelengths were obtained via the intersection of the straight-line extrapolations below and above the small photon energy knee, in the Tauc plots from the curves presented in Figure 5a, which provided the gap energy values presented in Table 1. Figure 5b presents the calculation of the band gap energies in the case of the pure TiO_2_ and TiO_2_ + N samples.

Ground state diffuse reflectance absorption studies for the nanopowders show that, while the N dopant causes deviations of the band gap to higher energies, doping TiO_2_ with Y suggests the opposite effect. Indeed, the value of energy gap ∆E increased from 3.13 eV (undoped) to about 3.16 eV in TiO_2_ doped with N and to 3.22 eV with 2% Y-N, and it reached 3.13 eV for 4% Y-N (Table 1). On the other hand, with 2% Y dopant, ∆E decreased to 3.10 eV, showing that Yttrium shifts the wavelength absorption range to higher values and this also explains the difference between 2% Y-N and 4% Y-N. The same trend was found in the work of Zhao and co-workers [23], which showed that Y-doping also induced a red shift of the absorption edge. Concretely, the absorption threshold shifted from 395 nm (undoped) to 405 nm (3% Y-TiO_2_). The most probable reason was that Y doping induced a part of the Y 4d states to extend into the TiO_2_ conduction band, resulting in a narrowing of the band gap of anatase. Therefore, these results suggest that the introduction of these two elements (N, Y) can modify the optical behavior of the material in different ways, presumably due to opposite functions related with the modification of the energy band gap and/or the creation of surface traps for the semiconductor’s charge carriers.

### 2.5. Laser Induced Luminescence Studies

**Figure 6:**
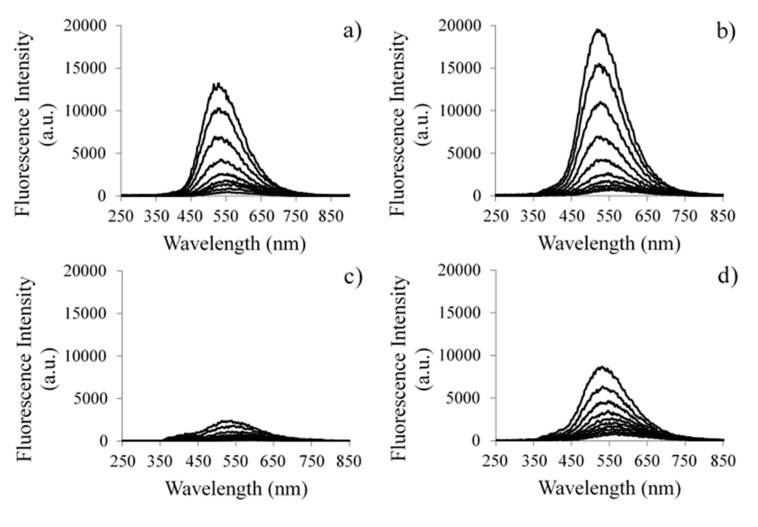

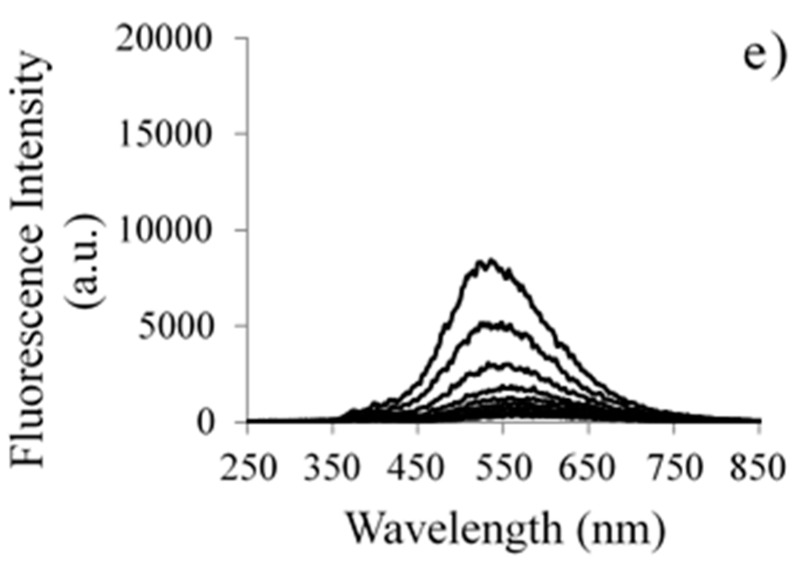
Time resolved laser induced luminescence spectra, at 77 K, of: anatase undoped (**a**); doped with N (**b**); doped with 2% Y (**c**); codoped with 2% (**d**) and 4% of Y-N (**e**). The excitation wavelength was 337 nm. The initial curve was obtained immediately after the laser pulse, and, for all of other curves, the time step was 20 ns annealed at 400 °C.

Figure 6a–e shows the laser induced luminescence spectra (λ_exc_ = 337 nm, ~1.3 mJ per excitation pulse), at 77 K of the powdered samples. The initial curve was obtained immediately after laser pulse, and the other curves are separated by steps of 20 ns. The used time gate width was one microsecond. The laser induced emission spectra of undoped TiO_2_ is presented in Figure 6a, with a maximum at 520 nm. It exhibits a decay with a lifetime of aproximately 59 ns. This luminescent emission is attributed to the typical and well-known green luminescent band of anatase. This emission occurs due to the recombination of the excited charge carriers, the electron and hole pair, from trap states located either at the surface or within the bulk phase of the crystalline material [24]. Figure 6b presents the luminescence spectra of TiO_2_, doped with N. The fluorescence lifetime of the sample changes, increasing to approximately 65 ns. It was previously mentioned that, with the introduction of N, the energy band gap increased, compared with the undoped TiO_2_. It seems that, as the energy bang gap is higher, so is the fluorescence lifetime. These results suggest that, together with the broadening effect of N observed in Raman spectra, the element could serve as surface defects, which can decrease the material’s crystallinity but simultaneously enhance the electron-hole separation, functioning in this case as trap sites for the separated charged states of the semiconductor. The results presented in Figure 6c prove that, as opposed to N-doped sample, Yttrium should enable the creation of intraband gap states that do not alter the crystallinity but actually change the electronic recombination mechanism of the semiconductor. In this case, a significant quenching of the fluorescence signal is observed, together with a small decrease of the fluorescence lifetime, which is now approximately 58 ns. This points out to a slight increase of the recombination rate of the electron-hole pair. Figure 6d,e confirm the trend, presenting a significant change in the fluorescence lifetime from the codoped 2% Y-N sample to the codoped 4% Y-N sample, which infers a decrease of approximately 59 ns to around 44 ns, respectively. Therefore, the photoluminescence of these samples is, as expected, directly correlated with the recombination process of the electron-hole pair [25]. Indeed, by increasing the energy gap and while delaying the recombination carrier, the radiative recombination lifetime should be higher, leading also to higher luminescence lifetimes. The doping of Yttrium into titania quenches the fluorescence signal because the excited electron can transfer from the valence band to the new levels that exist beneath the conduction band, which can decrease the photoluminescence intensity. In this way, TiO_2_ doped with N and co-doped with 2% Y-N could have a higher photo-catalytic activity by delaying the electron-hole recombination, despite their lower absorption in the visible range, when compared with the sample doped with 2% Y. However, it is important to be precise that the value of the lifetime of photoexcited charge in 2% Y-N is much smaller than the observed one in the 2% Li-N codoped sample, which is 72 ns [15].

### 2.6. Photocatalytic Degradation

**Figure 7:**
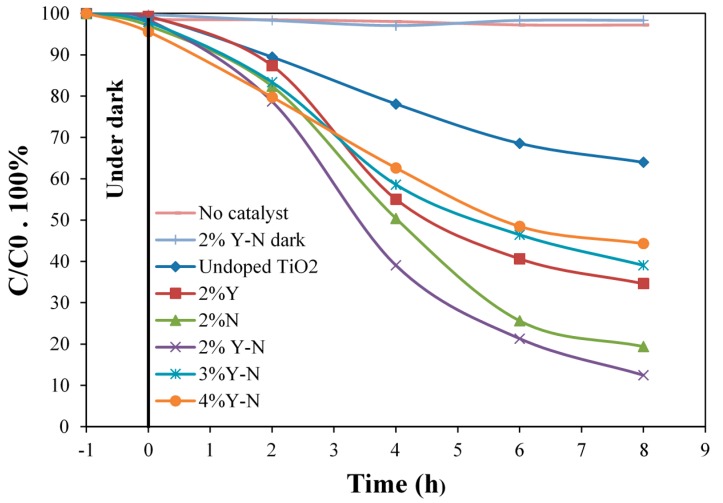
Evolution of the residual concentration of toluidine in the presence of undoped, Y-N doped and codoped TiO_2_ thin films annealed at 400 °C.

The photocatalytic activity of all prepared samples was evaluated by measuring the decomposition rate of toluidine under photoexcitation from a halogen light source. Figure 7 shows the degradation of the organic solute model without any catalyst and in the presence of undoped TiO_2_, N or Y doped and Y-N codoped TiO_2_ at a level of 2%. As shown in Figure 7, the residual concentration of toluidine after 8 h of irradiation in the presence of undoped TiO_2_ thin films calcined at 400 °C is about 64%, attesting the low photocatalytic activity of the pure TiO_2_. Doping TiO_2_ with N or Y notably affects the photocatalytic activity. Results reported in Figure 7 revealed different trends depending on the doping element. The largest catalytic effect is observed in the presence of 2% Y-N, for which the residual concentration of toluidine attained 12% after 8 h of light irradiation. One should note that, although all of the samples crystallize in the anatase form of TiO_2_, their photocatalytic activities differ considerably. This rules out the assignment of the difference in the degradation performance to the structural properties. However, this result is probably due to the synergetic role of the dopants dispersed on the TiO_2_ surface (as demonstrated by the decrease of crystallite sizes of the samples), yielding low average distances between the dopant centers acting as traps. However, compared to 2% Y-N codoped TiO_2_, increasing the Y level leads to huge decreases in the photocatalytic performance. Indeed, the residual concentration of toluidine reached 39% and 44% in the case of 3% Y-N and 4% Y-N, respectively. We argue that lower Y doping is more beneficial to the degradation process compared to higher Y doping. In addition, with the increase of the Y doping level, the distance between trapping sites decreases. The Y site at the surface of TiO_2_ nanoparticles can act as the charge carrier recombination center [17], which results in a decrease of the number of photogenerated charge, which contributes in the degradation process. Photoluminescence analysis corroborates well with these experimental observations, since the lifetime significantly decreases with increasing the doping level of Y from 2% Y-N to 4% Y-N.

In summary, careful analysis of the experimental results reveals energy gap values, which only allows the absorption of irradiation in the UV region. However, interesting performance was evidenced for the photodegradation of toluidine pollutant under halogen lamp, especially in the presence of 2% Y-N codoped TiO_2_. Both sunlight and W-Hal lamp have a small tail in the UV region, and this may explain the observed photocatalytic activity. To explain this trend, numerous hypotheses have been proposed, some relevant ones that claimed that the N dopant atoms resulted in the creation of surface oxygen vacancies (OVs) [26]. On the other hand, Y dopant seems to promote the recombination process, thereby decreasing the photocatalytic efficiency. This reactivity was also attributed by other authors to a charge-transfer-complex mechanism, in which neither the photocatalyst nor the organic compounds absorb visible light by themselves [27,28].

### 2.7. Reuse of the Photocatalyst

**Figure 8:**
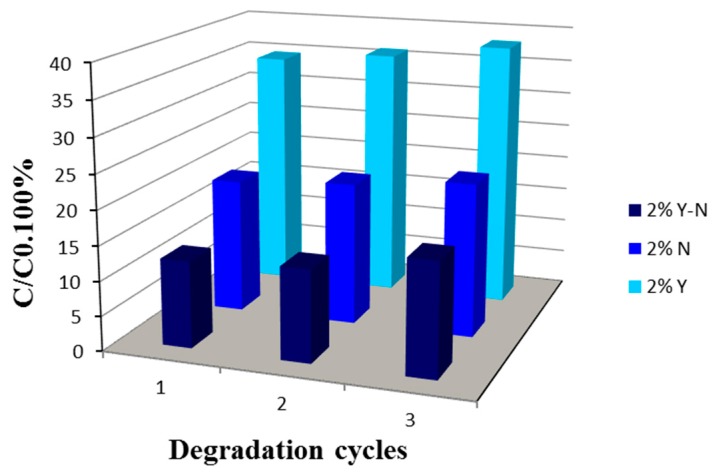
Evolution of the residual concentration of toluidine after three degradation cycles in the presence of 2% N, 2% Y and 2% Y-N codoped TiO_2_ photocatalyst annealed at 400 °C.

In view of practical application, the photocatalyst should be chemically and optically stable after several repeated cycles. To investigate the reusability of 2% N, 2% Y and 2% Y-N doped and codoped TiO_2_ in the photocatalytic degradation of toluidine experiment was repeated three times. The concentration of the organic pollutant was measured after 8 h exposed to lamp irradiation. After each decomposition reaction, the beads were rinsed with water, dried and used again in the same conditions. As shown in Figure 8, the better results were observed in the presence of 2% Y-N codoped TiO_2_, for which, after three cycles, a slight decrease in photodegradation efficiency was observed. In fact, in this case, the residual concentration of toluidine increased from 12% to 16%. This may be explained by the formation of some intermediate species that remained adsorbed at the surface of catalyst particles. Judging from these results, 2% Y-N codoped TiO_2_ can be regarded as stable and owing a considerable reproducibility of photodegradation of toluidine, which makes its use in the practical process possible. Of course, further work is required to better understand factors that affect the photodegradation rate of 2% Y-N codoped TiO_2_ and to further improve the photocatalyst stability.

## 3. Materials and Methods 

### 3.1. Preparation of Films and Powders

Y-N co-doped TiO_2_ samples were deposited using the sol–gel chemical process and dip-coating techniques with Ti(OBu)_4_ as precursor and acetylacetone (C_5_H_8_O_2_) as solvent. To achieve the doping, YCl_3_·6H_2_O and CH_3_(CH_2_)_4_CH_2_NH_2_ were used as sources of yttrium and nitrogen dopants, respectively. As a first step, an appropriate amount of YCl_3_·6H_2_O was dissolved in acetylacetone. Three different solutions corresponding to three atomic ratios Y/Ti = 0.02, 0.03 and 0.04 were elaborated. The hexylamine was then slowly added to the limpid solution under vigorous stirring (Atomic ratio N/Ti = 0.02). Afterwards, Ti(OBu)_4_ were introduced. Then, the activated substrate of glass was immersed in the prepared sol for 2 min. The obtained transparent thin films were then calcined at different temperatures: 400, 500, 550 and 650 °C for 2 h by heating at a rate of 5 °C/min followed by a free cooling. The remaining solutions were kept under stirring for several hours to obtain the powdered samples. For comparison purposes, undoped and Y or N monodoped TiO_2_ were prepared using the same synthetic protocol.

### 3.2. Physical and Chemical Characterization of the Catalysts

The surface morphology of the Y-, N-doped or codoped TiO_2_ thin films was studied by field-emission scanning electron microscopy (FE-SEM) using ZEISS SUPRA40 (ZEISS, Germany) fully controlled from a computer workstation. The electron source, a hot cathode producing electrons by Schottky effect, is a tungsten filament coated with a ZrO layer. Images are created by the software SMARTSEM.

The crystal structure of the obtained films was characterized by the grazing angle X-ray diffraction GXRD technique using an X-ray diffractometer Panalytical Xpert with CuKα radiation. The scanning range (2θ) was from 20° to 75° with a scanning rate of 0.05°/min and an incidence angle of 0.5°.

The Raman spectra of the powders were obtained in a back-scattering micro-configuration, with 532 nm excitation (Cobolt Samba CW DPSSL, 300 mW, Stockholm, Sweden) and by the use of a SuperHead 532 from Horiba JobinYvon (JY) (Villeneuve-d’Ascq, France) with a 50× Edmund long working distance objective. The Raman probe was coupled to a Shamrock 163 with a 100 µm entrance slit) and a Newton DU 971P-BV camera from Andor (Belfast, UK) was used as a detector for the Raman signals, working at −60 °C. Data acquisition was performed with the Andor software and data processing, namely, the baseline corrections, when needed, were made with the LabSpec software from JY (version 5.25.15, Villeneuve-d’Ascq, France). The spectral resolution of this Raman spectrometer was ~2 cm^−1^.

For the thin films, Raman spectra were recorded on a JYT64000 Raman spectrometer. A signal was obtained on excitation of the samples by an ArKr laser (514.5 nm). Measurement was done with the beam path set at (50×) and an exposure time of 2 × 300 s.

Ground-state absorption studies were performed using a homemade diffuse reflectance laser flash photolysis setup, with a 150 W tungsten-halogen lamp as monitoring lamp, triggering the system in the normal way but without the laser fire, and, in this way, recording the lamp profile for all samples under study and also for two standards, barium sulfate and magnesium oxide powders. A fixed monochromator coupled to an intensified charge coupled device (ICCD) with time gate capabilities was used for detecting the reflectance signals. The reflectance, R, from each sample was obtained in the UV–Vis-NIR (UV–Ultraviolet; Vis–visible; NIR-near infrared) spectral regions and the remission function, F(R), was calculated using the Kubelka–Munk equation for optically thick samples. The remission function is F(R) = (1 − R)^2^/2R. 

The set-up for time resolved luminescence LIL (laser induced fluorescence, LIF, and laser induced phosphorescence, LIP) is presented in Refs. [29,30]. Time-resolved emission spectra were performed in the nanosecond to second time range with an N^2^ laser (Photon technology international-PTI, model 2000, ca. 600 ps, full width half maximum (FWHM), about 1 mJ per pulse, 337 nm of excitation, PTI, ON, Canada).

### 3.3. Photocatalytic Activity Measurements

The photocatalytic performance of the Y-N codoped TiO_2_ was quantified by measuring the rate of degradation of organic solute model under halogen light irradiation. In a typical measurement, 25 mL of 5.10^−4^ mol L^−1^ solution of the organic solute was prepared. Titanium dioxide films deposed on beads of glass were introduced as photocatalysts. Prior to illumination, the suspension was stirred in the dark for 1 h to establish an adsorption/desorption equilibrium between the photocatalyst and pollutant molecules. Then, the photocatalytic degradation of pollutant model was initiated. The degradation test was carried out using a 47 W halogen lamp power (230 V), as a source of irradiation. The use of a lamp assures a constant light output in opposition to the fluctuation in solar intensity with season and time of day. To evaluate the organic solute concentration during the photodegradation reaction under constant irradiation, 1 mL of the suspension was taken out at a given time interval and the UV absorbance at their corresponding λ_max_ (at 280 nm) was measured. The concentration was evaluated from the calibration curve previously established with a known concentration of aliquot. 

## 4. Conclusions

In this work, Y-, N-doped or codoped TiO_2_ powders and thin films deposited on glass beads were successfully prepared using the sol-gel method, Ti (OBu)_4_ as Ti precursor. 

Structurally, the samples were found to be anatase with uniform shape nanoparticles (20–30 nm) as evidenced by FE-SEM observation. Ground state diffuse reflectance absorption studies showed that doping with N or codoping with N and Y led to an increase of the band gap. Laser induced luminescence analysis revealed a decrease in the recombination rate of the photogenerated holes and electrons. 

The photocatalytic activity of the prepared catalysts, under halogen light irradiation, was evaluated using toluidine as pollutant model. Results showed a great enhancement in the photocatalytic efficiency following the corporation of Y-N simultaneously, which is probably due to an electron-transfer mechanism, in which neither the photocatalyst nor the organic compounds absorb visible light by themselves. The photocatalyst was reusable for several degradation cycles with only a small reduction in the degradation efficiency. This advantage is important in the continuous photocatalytic degradation process.

## Figures and Tables

**Table 1:** Energy gaps and λ_abs_ for TiO_2_ undoped, doped and codoped with N and Y.

Sample	λ_abs_ (nm)	E_g_ (ev)
Undoped TiO_2_	396	3.13
N doped	392	3.17
2% Y doped	400	3.10
2% Y-N codoped	385	3.22
4% Y-N codoped	396	3.13
